# Fluorinated molecular beacons as functional DNA nanomolecules for cellular imaging†
†Electronic supplementary information (ESI) available. See DOI: 10.1039/c7sc02819a



**DOI:** 10.1039/c7sc02819a

**Published:** 2017-08-21

**Authors:** Cheng Jin, Ting Fu, Ruowen Wang, Hui Liu, Jianmei Zou, Zilong Zhao, Mao Ye, Xiaobing Zhang, Weihong Tan

**Affiliations:** a Molecular Science and Biomedicine Laboratory , State Key Laboratory of Chemo/Bio-Sensing and Chemometrics , College of Chemistry and Chemical Engineering , College of Life Sciences , Aptamer Engineering Center of Hunan Province , Hunan University , Changsha , Hunan 410082 , China . Email: tan@chem.ufl.edu ; Email: ruowenwang@ufl.edu; b Department of Chemistry , Department of Physiology and Functional Genomics , Center for Research at the Bio/Nano Interface , Health Cancer Center , UF Genetics Institute , McKnight Brain Institute , University of Florida , Gainesville , Florida 32611-7200 , USA; c Department of Biotechnology and Biomedicine , Yangtze Delta Region Institute of Tsinghua University , Zhejiang 314006 , China

## Abstract

Molecular beacons (MBs) are simple, but practical, fluorescent nanoprobes widely used to detect small molecules, nucleic acids and proteins. However, some challenges still remain when MBs are employed in complex biological environments, such as instability and non-target interference. To meet such challenges, we have designed and synthesized fluorinated molecular beacons (FMBs) as functional DNA nanomolecules for cellular imaging, in which the stem sequence is simply composed of artificial nucleotides with 3,5-bis(trifluoromethyl)benzene (F) as the surrogate base of natural A, T, C and G bases. The introduction of F base into MBs significantly increases their hydrophobicity, and the stem is formed by the assembly of self-complementary base F nucleotides through hydrophobic interactions. Fluorescence studies revealed that FMBs confer improved stability over conventional MBs. To demonstrate the application of FMBs for cellular imaging, we constructed an FMB to detect mRNA in MCF-7 cells, and the FMB was proven to be a practical nanoprobe for cellular imaging of mRNA.

## Abstract 2

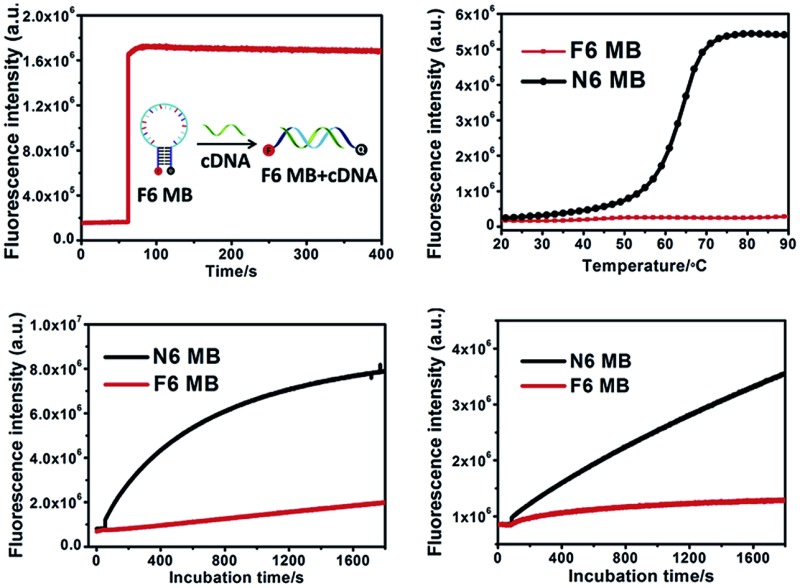
Molecular beacons (MBs) are simple, but practical, fluorescent nanoprobes widely used to detect small molecules, nucleic acids and proteins.

## Introduction

Live cells are defined by an intricate network of interacting genes, mRNAs, proteins and small molecules.^1–3^ To visualize and, hence, gain an understanding of intracellular interactions and processes, imaging techniques with high resolution and dynamic imaging capability are required.^4–7^ However, the cell cytoplasm is an unfriendly environment for probes. For example, intracellular enzymes can digest the probe, resulting in false-positive signals.^8,9^ Therefore, probes that are sensitive, but stable, are preferable for intracellular measurements.

DNA has been widely exploited as an intelligent and advanced material in construction of DNA-based nanodevices for biological applications. Molecular beacons (MBs) are dual-labeled nanomolecules with a fluorophore as a reporter at one end and a quencher at the other end.^10–16^ In the absence of targets, MBs form a hairpin structure by intramolecular hydrogen bond interaction of Watson–Crick bases, and the fluorescence is quenched by the close proximity of fluorophore and quencher. Compared to other fluorescent probes, MBs are highly sensitive for real-time monitoring based on their inherent fluorescence signal transduction mechanism. Another advantage of these nanoprobes is specific target recognition based on their loop and stem structure. The simple but practical architecture of MBs has resulted in widespread applications that range from the detection of single-nucleotide polymorphisms (SNPs) to bacterial bioterrorism agents.^17–22^

However, some challenges still remain when MBs are employed in complex biological environments, especially in living cells. The hairpin structure of an MB is often disrupted by the interaction of stem sequences with nontarget small molecules, proteins or nucleic acids, thus giving false-positive signals.^23,24^ The closed stem structure may also be opened in response to an acidic environment or temperature changes.^25^ MBs composed of natural bases are not stable against nucleases and cell lysate, and such degradation in living cells also gives false-positive signals.^26^

To address such challenges, researchers have developed advanced MBs by introducing artificial nucleosides. For instance, to enhance thermostability of MBs, locked nucleic acid/base pairs have been modified in the stem region to allow a constant low fluorescence background. To reduce false-positive signals and improve specificity and stability of MBs, artificial nucleoside pairs Z and P have been encoded into MBs to resist degradation by enzymes.^27–32^

**Scheme 1:**
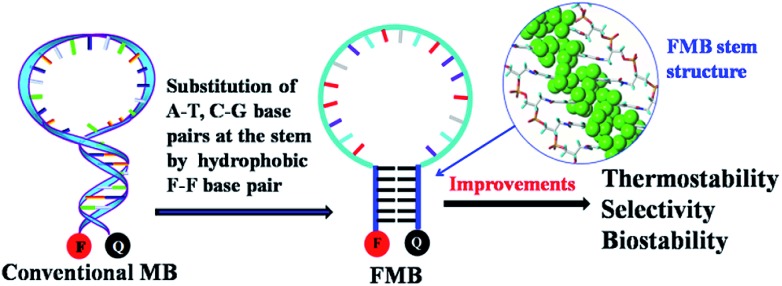
The design of FMBs as functional nanomolecules and their potential improvements over conventional MBs.

In a previous work, we incorporated 3,5-bis(trifluoromethyl)benzene as a hydrophobic base into oligonucleotides and found that a stable planar duplex was formed merely through F–F base pairing.^33^ Based on this observation, we hypothesized that an MB constructed through hydrophobic F–F base pairing would present the following advantages over conventional MBs: (1) enhanced stability against enzymatic degradation by the introduction of artificial base F; (2) resistance to interference with other biomolecules by self-assembly of the stem through hydrophobic interactions instead of hydrogen bonding (Scheme 1). Artificial DNAs may provide promising solutions for challenges strangling wide applications of DNA nanoprobes and nanodevices, and thus we illustrate here the construction of molecular beacons with hydrophobic bases for intracellular imaging.

## Results and dissusion

### Formation of beacon structure

**Fig. 1:**
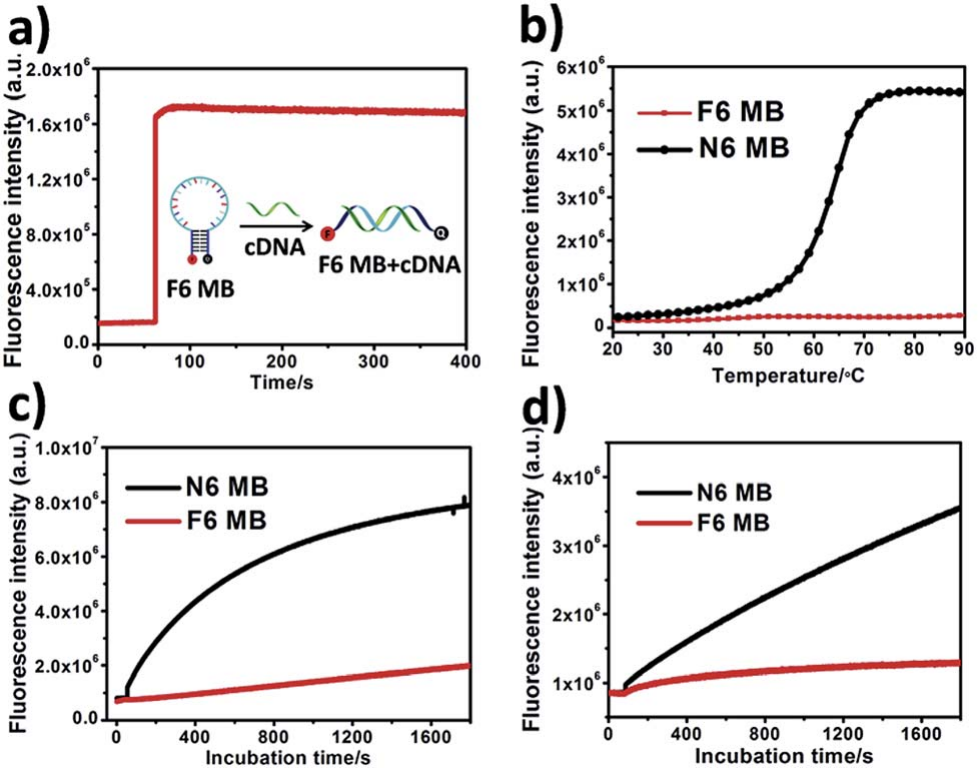
Properties of F6 MB. (a) Fluorescence spectroscopy kinetics of F6 MB treated with cDNA. (b) Melting curves for F6 MB and N6 MB. Both MBs were diluted to 200 nM and incubated with PBS buffer, followed by the melting curve test. Temperature was brought to 20 °C and increased at 1 °C increments per minute to 90 °C. (c & d) Fluorescence spectroscopy kinetics of F6 MB (red) and N6 MB (black) treated with 0.25 U mL^–1^ DNase I endonuclease (c) and CCRF-CEM cell lysate (d).

To test our hypothesis, a series of trifluoromethylated oligonucleotide molecular beacons (Table S1†) were readily synthesized from our previously developed phosphoramidite F. Conventional MBs with six natural base pairs in the stem region (N6 MBs) and a series of oligonucleotides were also synthesized for the investigation. Both F4 MB (four F–F base pairs in the stem, see Table S1† for detailed sequence) and F6 MB (six F–F base pairs in the stem, see Table S1† for detailed sequence) form stable hairpin structure similar to conventional MBs. In comparison, F6 MB displays better signal-to-background ratio than F4 MB (Fig. 1a and S1†), and a somewhat better discriminatory power to a single-base mismatch of the target DNA sequence compared to N6 MB (Fig. S2†).

Thermodynamics study. Conventional MBs maintain their closed hairpin conformation at room temperature. However, with increasing surrounding temperature, conventional MBs slowly change their conformation to the opened state, resulting in enhanced background fluorescence intensity (Fig. 1b, black line). As shown in Fig. S4,† the S/B ratio of N6 MB was about 12.6-fold at 20 °C, 11.3-fold at 30 °C and only 8.4-fold at 37 °C. Given preferable and reproducible detection under dynamic temperature change within the scope of room temperature, novel MB-based nanoprobes with better thermostability would provide more attractive performance in analytical detection.^28^ Interestingly, unlike N6 MB, F6 MB maintained its conformation even at 90 °C (Fig. 1b, red line) with constant S/B ratios of 10.6-fold at 20 °C, 10.6-fold at 30 °C and 10.0-fold at 37 °C (Fig. S4†). This phenomenon is explained by the following points. First, F–F base pairs display stronger binding free energies than natural base pairs,^33^ providing tighter interaction in the stem region. Second, hydrophobic interactions between fluorinated base pairs show stronger resistance against temperature change compared to hydrogen bonding between natural base pairs.^34,35^ We further verified that base pairing between F base pairs contributes to the super-thermostability of FMBs. To prove this, a molecular beacon combining six natural base pairs and six fluorinated base pairs in the stem region was synthesized and termed F6N6 MB. F6N6 MB also shows thermostability in high surrounding temperatures (Fig. S6†). Taken together, this evidence proved the super-thermostability of FMBs and, in particular, revealed that F6 MB could detect targets under relatively severe changes in ambient temperature.

### Enhanced biostability

Intracellular biodegradation is a key factor affecting intracellular application of MB-based nanoprobes, as it can also result in false-positive signals. Therefore, we next investigated the resistance of F6 MB to digestion by endonuclease and cell lysate compared with N6 MB. As shown in Fig. 1c, a significant fluorescence increase of N6 MB at about 8.9-fold in 30 minutes was observed after the addition of 0.25 U mL^–1^ DNase I, indicating that N6 MB suffered severely from enzymatic digestion. In contrast, F6 MB showed better resistance to enzymatic digestion of about 2.5-fold in 30 minutes. When stability against cell lysate was tested, results showed that F6 MB had better resistance to digestion compared to N6 MB (1.5-fold of F6 MB *vs.* 4.2-fold of N6 MB) (Fig. 1d). Compared with natural base pairs, fluorinated base pairs cannot be recognized by endonuclease. Consequently, the replacement of natural base pairs by fluorinated base pairs makes F6 MB more resistant to biodigestion compared to N6 MB. Responses of both MBs to cDNA in cell lysate solution were investigated to determine their practical utility in complex *in vitro* systems. Because of severe digestion by cell lysate, N6 MB only had about 2.6-fold fluorescence enhancement after 30 minutes of incubation. For F6 MB, on the other hand, fluorescence enhancement was about 5.8-fold in the same conditions (Fig. S7†). This line of evidence demonstrates that F6 MB has better stability and, as a result, better fluorescence recovery in complex intracellular biological environments.

### Enhanced selectivity of FMBs

**Fig. 2:**
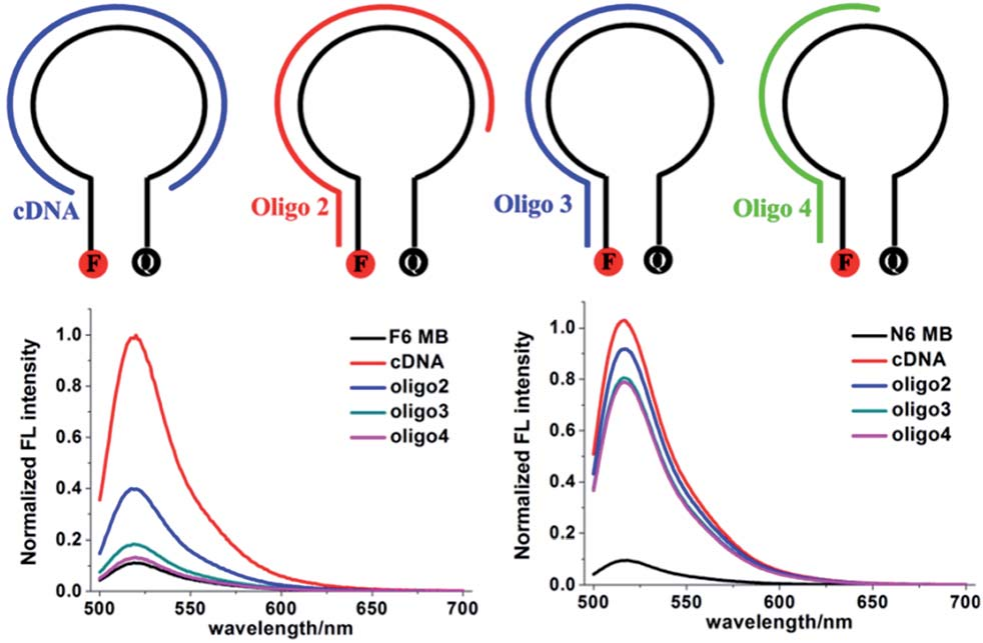
Normalized S/B fluorescence of F6 MB (bottom left) and N6 MB (bottom right) in PBS buffer. Final concentration ratio of F6 MB (or N6 MB): cDNA (or Oligo 2, Oligo 3, Oligo 4) = 1 : 1.

Next we compared the performance of F6 MB and N6 MB in the presence of nontarget nucleic acids. Fig. 2 shows signal enhancement after hybridization of N6 MB and F6 MB with cDNA, Oligo 2, Oligo 3 and Oligo 4 in PBS buffer. The S/B ratios of N6 MB and F6 MB are both approximately 10-fold when they hybridize with cDNA. In the presence of nontarget nucleic acids containing sequences complementary to the stem sequence of N6 MB, such as Oligo 4, Oligo 3 and Oligo 2, the S/B ratios of N6 MB range from 8.2-fold to 9.6-fold, suggesting that false-positive signals could result from interference with nontarget sequences. Compared with N6 MB, only negligible signal enhancements were observed when F6 MB hybridized with Oligo 3 or Oligo 4. For example, the signal enhancement was under 3.6-fold for F6 MB hybridized with Oligo 2. These fluorescence results verify that F6 MB is more resistant to interferences in complex biological environments than N6 MB.

### Intracellular mRNA imaging

**Fig. 3:**
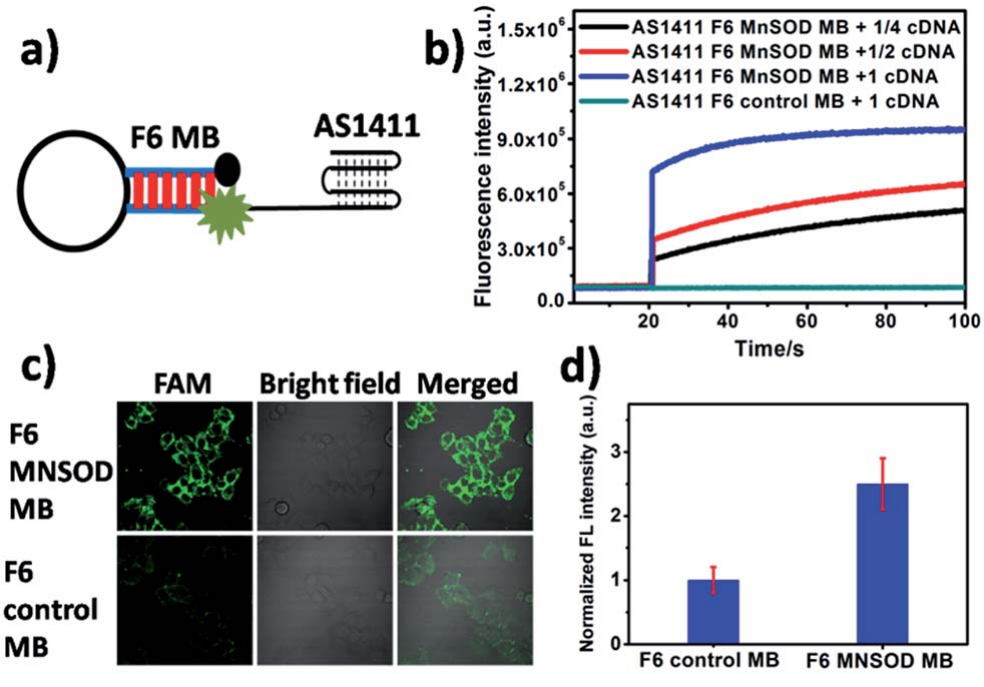
Schematic illustration of AS1411-linked F6 MnSOD MBs for intracellular MnSOD mRNA imaging. (a) Diagram of AS1411-linked F6 MnSOD MB. (b) Fluorescence spectroscopy kinetics of AS1411 F6 MnSOD MBs and AS1411 F6 control MBs treated with MnSOD DNA. (c) Confocal fluorescence imaging of LPS-pretreated MCF-7 cells incubated with fluorinated molecular beacon probes. (d) Relative fluorescence of MCF-7 cells incubated with F6 MnSOD MB and F6 control MB.

In addition to their degradation by nucleases, the cellular applications of MB-based nanoprobes, as oligonucleotides, are limited by their poor uptake by cells. For efficient delivery, MBs are typically injected into cells by microinjection technology or by complex delivery systems. In this paper, an extended internalized DNA strand AS1411 was linked at the terminus of F6 MB. AS1411, as a G-rich oligonucleotide, is capable of uptake into cytoplasm by the cell. The cellular internalization pathway was proved by a macropinocytosis process under the stimulation by a nucleolin-dependent mechanism. Because of its excellent target and internalization abilities to specific cancer cells, AS1411 and conjugated nanoparticles have been used widely in preclinical cancer diagnosis and therapy. Manganese Superoxide Dismutase (MnSOD) mRNA, which is overexpressed in MCF-7 breast cancer cells,^36–38^ was used as a model target in our cellular imaging study. AS1411-linked F6 MnSOD MB effectively recognized the target DNA sequence, providing about 10-fold fluorescence recovery in tube solution (Fig. 3b). AS1411-linked F6 MnSOD MBs with moderate concentration were then incubated with MCF-7 cells pretreated with lipopolysaccharide (LPS, 10 μg mL^–1^), followed by confocal fluorescence microscopy imaging. As is shown in Fig. 3c and d, an obvious fluorescence response (2.5-fold compared to control molecular beacon) was found after one hour incubation. However, only negligible fluorescence was observed for the same cells incubated with F6 control MB under the same conditions. These imaging results demonstrated that F6 MB is capable of imaging mRNA in the intracellular environment. To further assess the selectivity of AS1411-linked F6 MnSOD MBs to its targeted nucleic acids in cells, comparison of probes-incubated MCF-7 cells with and without mRNA overexpression was performed. As shown in Fig. S8,† an obvious fluorescence shift was observed in the case of LPS-treated cells (about 1.7-fold in intensity) compared to the non-overexpressed cells. This result provided an additional proof of the specific recognition of F6 MB to its target in cells. Next, using different DNA concentrations, a cell cytotoxicity assay was performed to determine cell viability of MCF-7 cells after binding with fluorinated DNA hairpin. Results indicated that the F6 DNA hairpin had almost no effect on cell proliferation, thus demonstrating its biosafety (Fig. S9†).

## Experiments section

### DNA synthesis

All DNA strands used in this work were synthesized on the Polygen 12-column DNA/RNA solid synthesizer on a 1.0 micromolar scale, using the corresponding controlled pore glass (CPG). As-synthesized 3,5-bis(trifluoromethyl)benzoyl phosphoramidite monomer was dissolved in anhydrous dichloromethane in 0.1 M concentration and coupled on the synthesizer with 600 s coupling time. After synthesis, the obtained oligonucleotides were cleaved and deprotected from the CPG, followed by precipitation in cold ethanol solution at –20 °C overnight. After centrifugation to remove supernatant solution, the DNA products were dissolved with 0.1 M TEAA and purified by reversed phase HPLC using a BioBasic4 column. Finally, the 4,4′-dimethytribenzyl group was removed from DNA by adding 80% acetic acid aqueous solution, and the DNA was again precipitated in cold ethanol. After drying in vacuum and the desalting step, the synthesized oligonucleotides were quantified by measuring their absorbance at 260 nm.

### Fluorescence measurements

A JY fluorimeter was used for all fluorescence measurements. For FAM group, the solution was excited at 488 nm and scanned from 500 to 700 nm with a bandwidth of 5 nm. For the kinetic mode of fluorescence measurements, the excitation wavelength was 488 nm, and emission wavelength was 520 nm with a bandwidth of 5 nm.

### Melting curve measurement

All measurements were performed in an Applied-Biosystems 7500 RT-PCR instrument with a sample concentration of 200 nM. The temperature was increased from 20 to 90 °C in 1 degree increments per minute. The FAM fluorescence signal was measured.

### Cell culture

MCF-7 cells were maintained in DMEM medium supplemented with 10% fetal bovine serum and 0.5 mg mL^–1^ penicillin–streptomycin at 37 °C in 5% CO_2_. CCRF-CEM (human leukemia) cells were maintained in 1640 medium, supplemented with 10% fetal bovine serum and 0.5 mg mL^–1^ penicillin–streptomycin at 37 °C in 5% CO_2_.

### Cell lysate preparation

About three million CCRF-CEM cells were washed twice with DPBS buffer and then dispersed in 1 mL DPBS buffer solution. The mixture was lysed with a sonicator for 2 minutes, and the resulting cell lysate solution was used immediately.

### Cytotoxicity assay

The cytotoxicity of fluorinated molecular beacons was evaluated using a 96-well proliferation assay at 37 °C in 5% CO_2_ atmosphere. A sample of 5000 MCF-7 cells in 100 μL fresh cell culture medium was seeded into each test well. After 24 h, cell culture medium was removed, and then another 100 μL of fresh culture medium without serum was added, followed by adding fluorinated DNA molecular beacons at the desired concentration. After 6 h treatment, culture medium without serum was removed and 100 μL of fresh cell culture medium was added for an additional 48 h treatment. Finally, 10 μL of CCK-8 was added to the well, and the 96-well plate was subjected to absorption measurement at 450 nm using a microplate reader.

### FACS analysis

MCF-7 cells were placed in a 35 mm cell culture dish and grown to around 80% confluence for 48 h before the experiment. Before incubation with molecular beacons, MCF-7 cells were pretreated with or without 10 μg mL^–1^ LPS (lipopolysaccharide) for 30 minutes. Then the solution was removed and cells were allowed to incubate with probes for one hour. After incubation, cells were washed twice with PBS buffer and then subjected to FACS assay.

### Confocal fluorescence microscopy imaging

MCF-7 cells were placed in a 35 mm cell culture dish and grown to around 80% confluence for 48 h before the experiment. Cells were washed with 1 mL PBS buffer containing 5 mM MgCl_2_. Before incubation with molecular beacons, MCF-7 cells were pretreated with 10 μg mL^–1^ LPS for 30 minutes. Then LPS solution was removed by washing with PBS. Treated cells were incubated with molecular beacon probes at the desired concentration in PBS buffer containing 5 mM MgCl_2_ and 4.5 mM glucose for one hour at 37 °C in 5% CO_2_. After incubation, cells were washed twice with PBS buffer and then subjected to confocal microscopy imaging.

## Conclusions

In summary, this study illustrates the synthesis and characterization of FMB-based nanoprobes. The introduction of the base surrogate F into MBs significantly increases their thermostability and improves stability against cell lysate over N6 MB. By imaging mRNA in MCF-7 cells with F6 MnSOD MB, we demonstrated the potential of this nanoprobe for dynamic monitoring of cell-specific targets.

## Conflicts of interest

The authors declare no competing financial interests.
