# Supplementary material for: Fluorinated molecular beacons as functional DNA nanomolecules for cellular imaging
Source: Chem Sci. 2017 Aug 21;8(10):7082–6. doi: 10.1039/c7sc02819a (PMC5637457; doi:10.1039/c7sc02819a)
Supplement: Supplementary file 1 [file SC-008-C7SC02819A-s001.pdf]

Electronic Supplementary Material (ESI) for Chemical Science.
This journal is © The Royal Society of Chemistry 2017

Supporting Information

Fluorinated molecular beacons as functional DNA nanomolecules for cellular imaging

Cheng Jin,[a,†] Ting Fu,[a,†] Ruowen Wang,[a, b, c] Hui Liu,[a] Jiamei Zou,[a] Zilong Zhao,[a] Mao Ye,[a] Xiaobing Zhang[a] and Weihong Tan[a, b]

a) Molecular Sciences and Biomedicine Laboratory, State KeyLaboratory for Chemo/Biosensing and Chemometrics, College of Chemistry and Chemical Engineering and College of Biology, Collaborative Innovation Center for Chemistry and Molecular Medicine, Hunan University, Changsha 410082, China

b) Department of Chemistry and Department of Physiology andFunctional Genomics, Center for Research at the Bio/Nano Interface, Health Cancer Center, UF Genetics Institute and McKnight Brain Institute, University of Florida, Gainesville, Florida 32611-7200, United States

c) Department of Biotechnology and Biomedicine, Yangtze DeltaRegion Institute of Tsinghua University, Zhejiang, 314006, China

[†]These authors contributed equally to this paper.

Correspondenceauthors: W.T. (tan@chem.ufl.edu) and R.W. (wangwrw@yahoo.com).

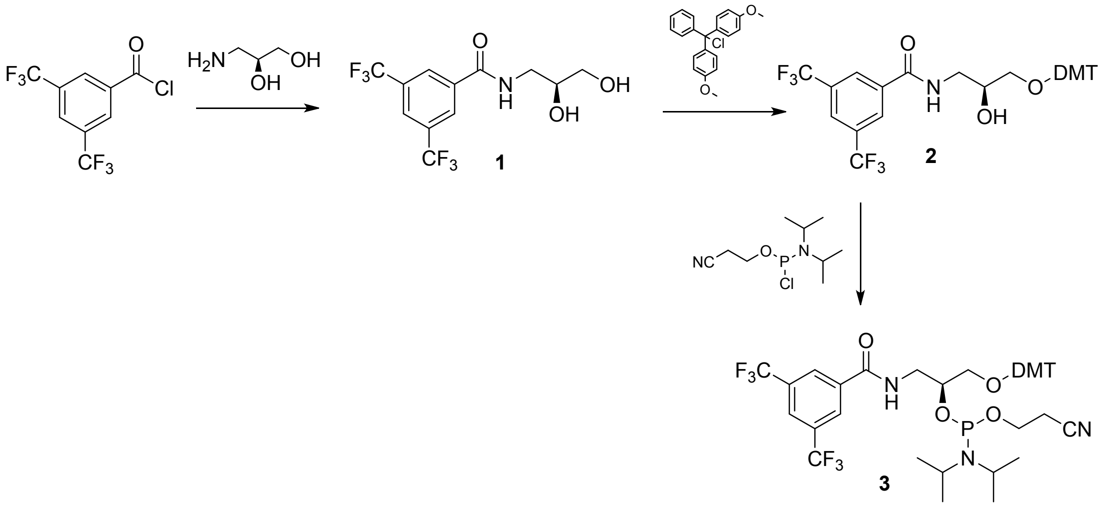

Scheme S1. Synthesis route of F base phosphoramidite

The synthesis procedures of fluorinated base phosphoramidite monomer refer to the protocol developed in our laboratory.[1] ^{1}HNMR, ^{19}FNMR, ^{31}PNMR and ESI-MS data of 3,5-bis(trifluoromethyl) benzoyl phosphoramidite monomer (3) are as follows:

^{1}H NMR (300 MHz, $CDCl_3$) δ: 8.43 (s, 1H), 8.41 (s, 1H), 8.23 (s, 1H), 8.17 (s, 1H), 7.51 (d, J=6.0 Hz, 2H), 7.37 (d, J=6.6 Hz, 4H), 7.26-7.30 (m, 2H), 7.187.21 (m, 1H), 6.83-6.87 (m, 4H),4.29-4.35 (m, 1H), 3.62-3.93 (m, 12H), 3.17-3.34 (m, 2H), 2.70-2.74 (m, 1H), 2.60-2.63 (m, 1H), 1.10-1.25 (m, 12H); ^{19}F NMR ($CDCl_3$) δ: -64.23, -64.29; ^{31}P NMR ($CDCl_3$) δ: 139.62, 140.99. MS (ESI+): m/z 833.3 (Calculated M+Na: 856.40).

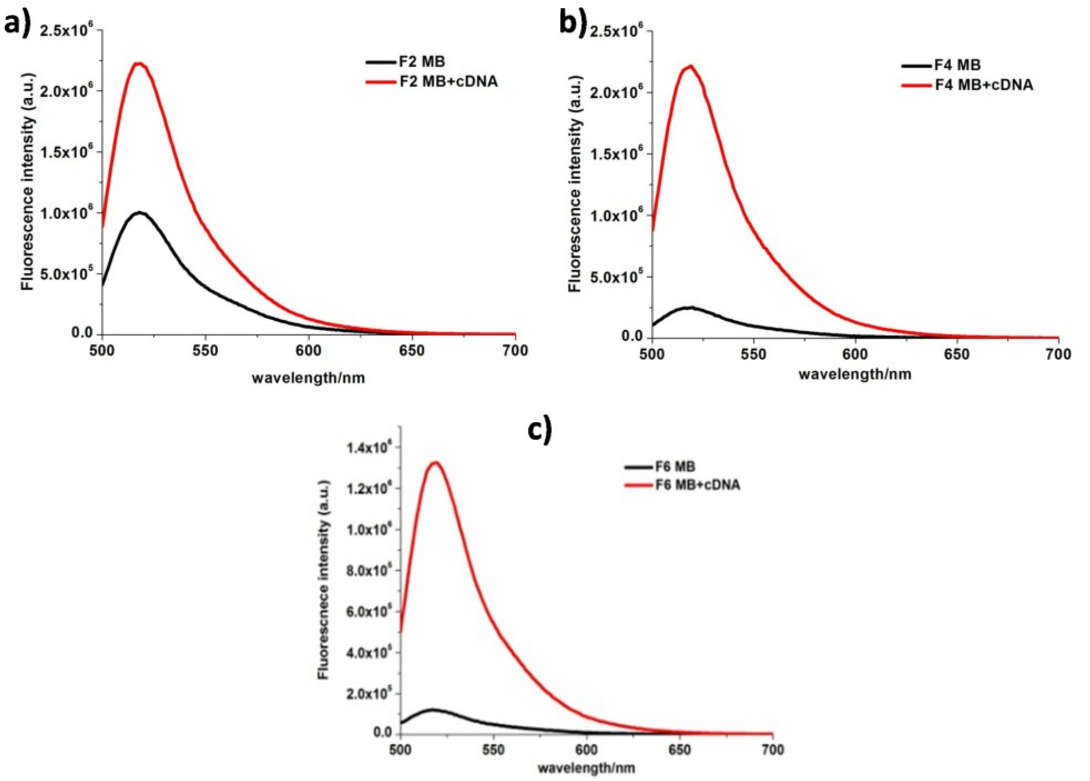

Figure S1. Fluorescence spectroscopy of FMBs before (black line) and after (red line) hybridization with cDNA. (a) F2 MB, (b) F4 MB and (c) F6 MB.

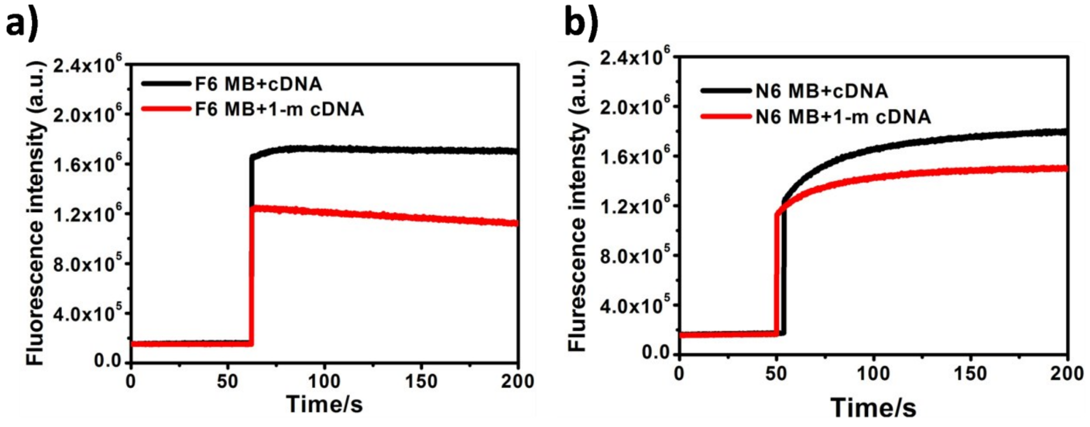

Figure S2. Fluorescence spectroscopy kinetics of F6 MB (a) and N6 MB (b) treated with cDNA (black line) and synthetic one-base mismatch target (1m-cDNA) (red line).

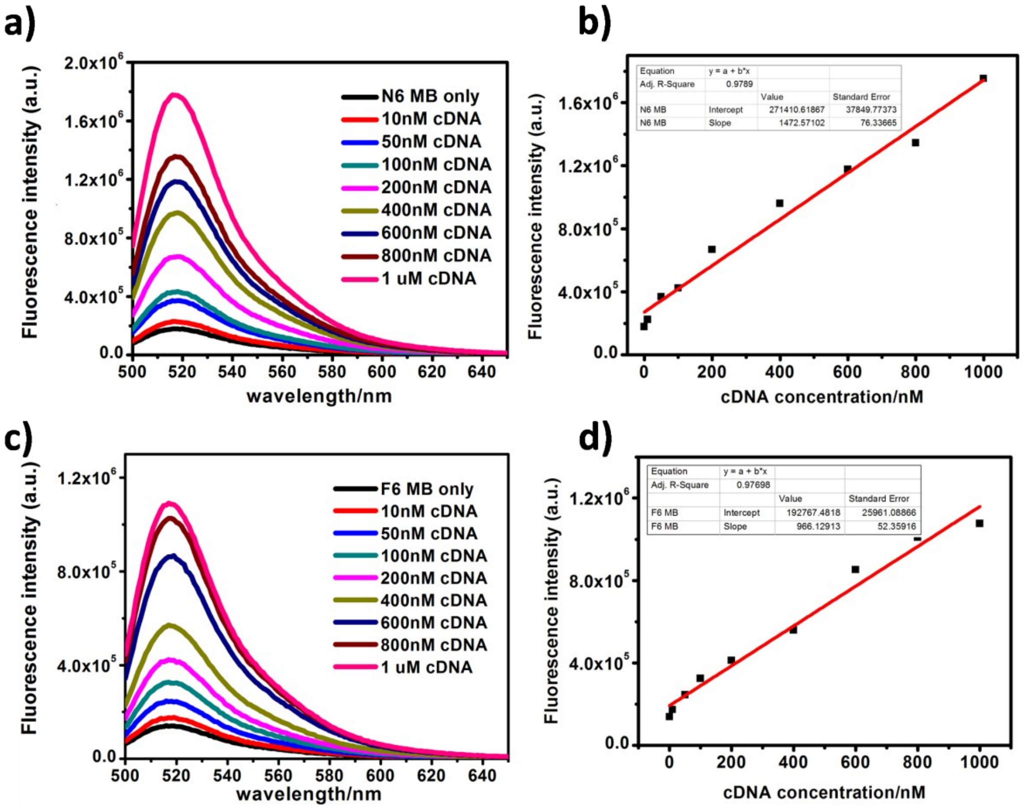

Figure S3. Fluorescence spectroscopy of 1 μM N6 MB (a) and F6 MB (c) treated with cDNA. b & d) The fitted linear relation of N6 MB (b) and F6 MB (d) function to the concentration of cDNA. In both cases, the lowest detectable concentration of target DNA is 50 nM.

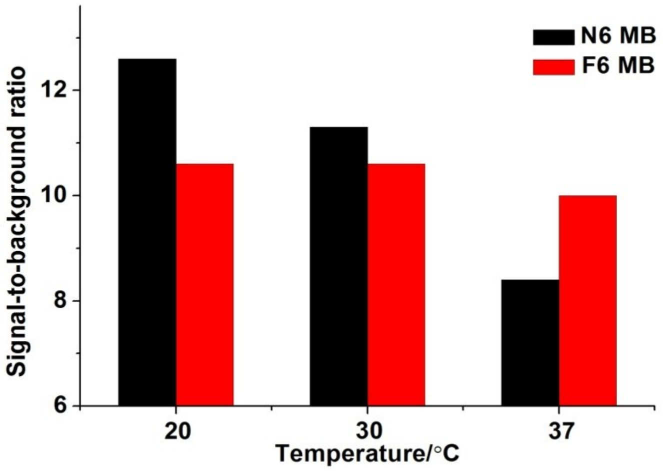

Figure S4. S/B ratios of F6 MB (red column) and N6 MB (black column) hybridized with cDNA at 20, 30 and 37°C.

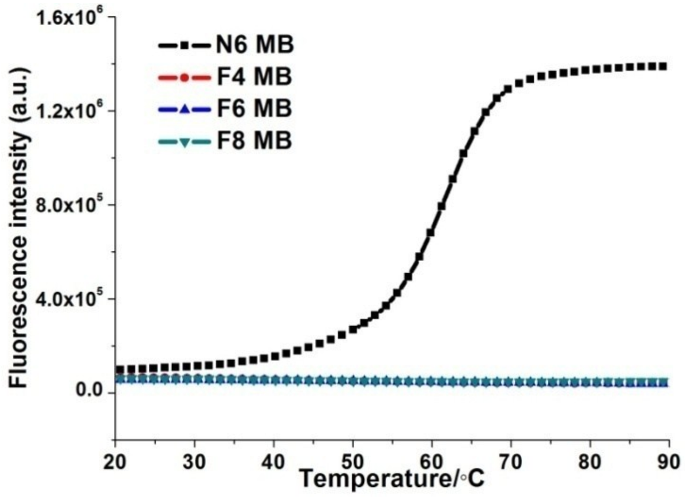

Figure S5. Melting curves of 200 nM FMBs and N6 MBs.

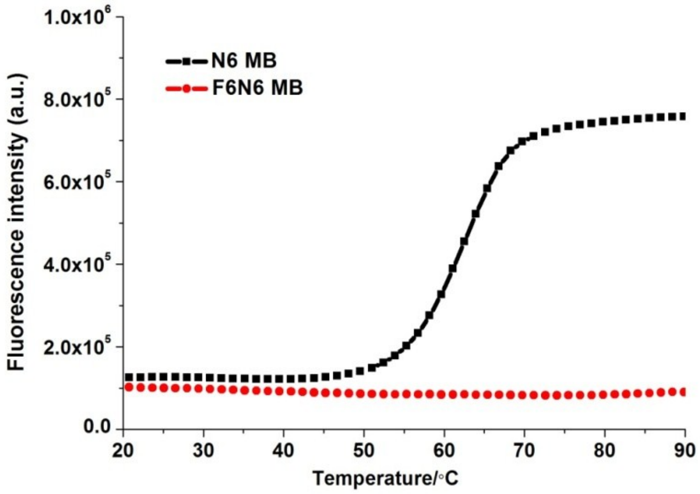

Figure S6. Melting curves of F6N6 MB and N6 MB. F6N6 MB represents FMBs with additional six natural base pairs in stem region.

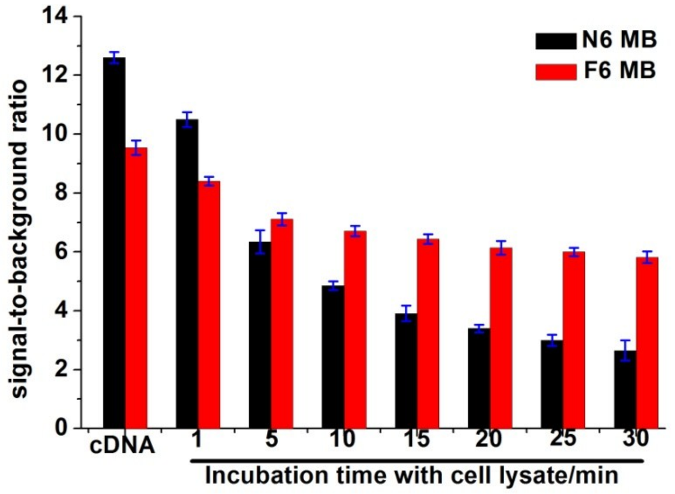

Figure S7. Comparison between the S/B ratio of F6 MB (red column) and N6 MB (black column) with various incubation times to CCRF-CEM cell lysate.

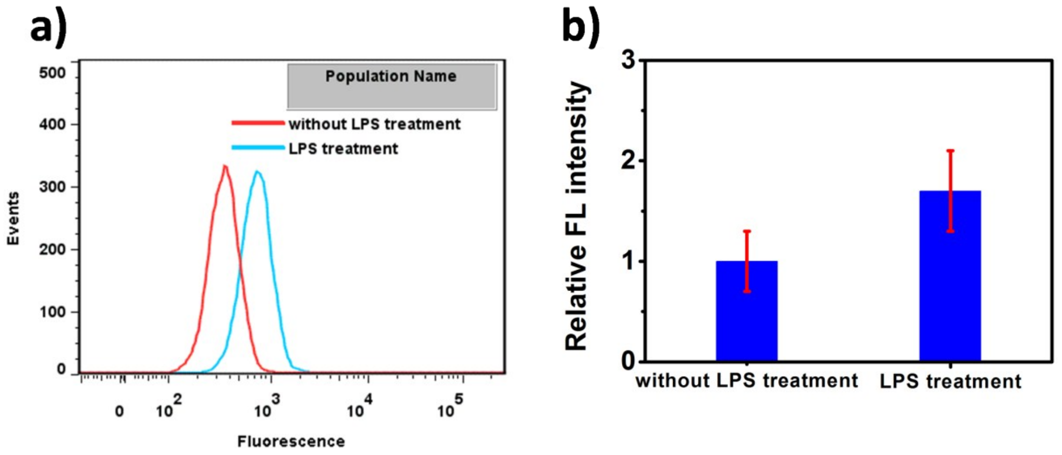

Figure S8. (a) Flow cytometry analysis and (b) the relative fluorescence intensity of MCF-7 cells (with and without LPS treatment) after incubation with AS1411-linked F6 MnSOD MBs.

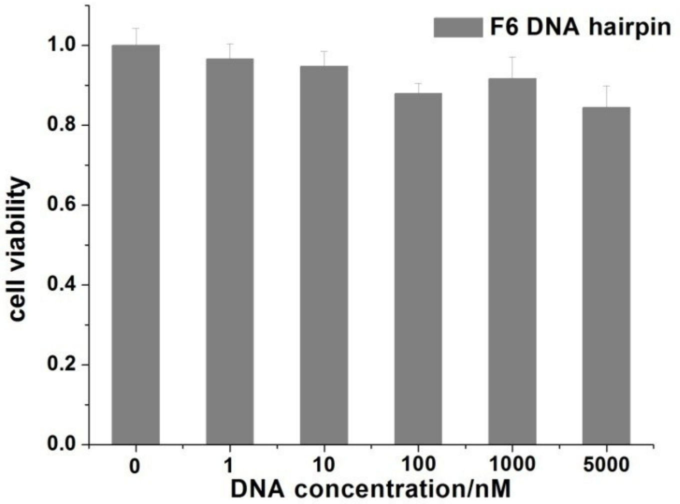

Figure S9. Cytotoxicity assay of MCF-7 cells treated with F6 DNA hairpin.

Table S1. DNA sequences designed in this article.

Name	Sequence (from 5'to 3')
F2 MB	FAM **FF** TCT AAA TCA CTA TGG TCG C **FF** Dabcyl
F4 MB	FAM **FFFF** TCT AAA TCA CTA TGG TCG C **FFFF** Dabcyl
F6 MB	FAM **FFFFFF** TCT AAA TCA CTA TGG TCG C **FFFFFF** Dabcyl
F8 MB	FAM **FFFFFFFF** TCT AAA TCA CTA TGG TCG C **FFFFFFFF** Dabcyl
F6N6 MB	FAM **FFFFFF CCG AGC** TCT AAA TCA CTA TGG TCG C **GCT CGG FFFFFF** Dabcyl
N6 MB	FAM **CCG AGC** TCT AAA TCA CTA TGG TCG C **GCTCGG** Dabcyl
cDNA	GCG ACC ATA GTG ATT TAG A
1m-cDNA	GCG ACC ATA G**A**G ATT TAG A
Oligo 2	CCA TAG TGA TTT AGA GCT CGG
Oligo3	TAG TGA TTT AGA GCT CGG
Oligo4	TGATTT AGA GCT CGG
AS1411-F6MnSOD MB	Dabcyl **FFFFFF** AGT TAC ATT CTC CCA GTT GAT T **FFFFFF** FAM TTT GGT GGT GGT GGT TGT GGT GGT GGT GGT TT
AS1411-F6 control MB	Dabcyl **FFFFFF** TCT AAA TCG CTA TGG TCG C **FFFFFF** FAM TTT GGT GGT GGT GGT TGT GGT GGT GGT GGT TT

MnSOD DNA	AAT CAA CTG GGA GAA TGT AAC T

Note: **F** refers to F base

Reference

1. R. Wang, C. Wang, Y. Cao, Z. Zhu, C. Yang, J. Chen, F.-L. Qing and W. Tan, *Chemical Science*, 2014, **5**, 4076-4081.
